# 3, 4-dihydroxy-L-phenylalanine-derived melanin from *Yarrowia lipolytica* mediates the synthesis of silver and gold nanostructures

**DOI:** 10.1186/1477-3155-11-2

**Published:** 2013-01-30

**Authors:** Mugdha Apte, Gauri Girme, Ashok Bankar, Ameeta RaviKumar, Smita Zinjarde

**Affiliations:** 1 Institute of Bioinformatics and Biotechnology, University of Pune, Pune 411 007, India

**Keywords:** *Yarrowia lipolytica*, L-DOPA melanin, Nanoparticles, Anti-fungal activity

## Abstract

**Background:**

Nanobiotechnology applies the capabilities of biological systems in generating a variety of nano-sized structures. Plants, algae, fungi and bacteria are some systems mediating such reactions. In fungi, the synthesis of melanin is an important strategy for cell-survival under metal-stressed conditions. *Yarrowia lipolytica*, the biotechnologically significant yeast also produces melanin that sequesters heavy metal ions. The content of this cell-associated melanin is often low and precursors such as L-tyrosine or 3, 4-dihydroxy-L-phenylalanine (L-DOPA) can enhance its production. The induced melanin has not been exploited for the synthesis of nanostructures. In this investigation, we have employed L-DOPA-melanin for the facile synthesis of silver and gold nanostructures. The former have been used for the development of anti-fungal paints.

**Methods:**

*Yarrowia lipolytica* NCIM 3590 cells were incubated with L-DOPA for 18 h and the resultant dark pigment was subjected to physical and chemical analysis. This biopolymer was used as a reducing and stabilizing agent for the synthesis of silver and gold nanostructures. These nanoparticles were characterized by UV-Visible spectra, X-ray diffraction (XRD) studies, and electron microscopy. Silver nanoparticles were evaluated for anti-fungal activity.

**Results:**

The pigment isolated from *Y. lipolytica* was identified as melanin. The induced pigment reduced silver nitrate and chloroauric acid to silver and gold nanostructures, respectively. The silver nanoparticles were smaller in size (7 nm) and displayed excellent anti-fungal properties towards an *Aspergillus* sp. isolated from a wall surface. An application of these nanoparticles as effective paint-additives has been demonstrated.

**Conclusion:**

The yeast mediated enhanced production of the metal-ion-reducing pigment, melanin. A simple and rapid method for the extracellular synthesis of nanoparticles with paint-additive-application was developed.

## Background

Nanotechnology is an inter-disciplinary science that involves the production, manipulation and use of materials in the nano-scale range. In recent times, “Nanobiotechnology” has emerged as an important branch of nanotechnology [1]. The combination of biological principles with physical and chemical processes generates specific nano-sized structures. Nanobiotechnology has played an important role in providing eco-friendly alternative routes for synthesizing nanoparticles [2,3]. Biosynthetic methods exploit the bio-metal interactive capabilities of plants and microorganisms [4-6]. The latter group includes a large plethora of microbial forms that mediate the synthesis of nanostructures in a very specific manner [7].

Microbial forms are constantly exposed to different metals and metalloids in the environment. Some of the metals are necessary for their survival while others are detrimental [8]. Bacteria and fungi adapt themselves to the presence of such metals via processes such as biosorption, bioprecipitation, extracellular sequestration and/or chelation [9]. Fungal melanins play an important role in enhancing cell-survival under conditions of metal-stress [10-12] since they possess several metal-binding sites [13,14].

In the biotechnologically important yeast *Y. lipolytica*, melanin is known sequester metal ions [15]. The content of the cell-associated melanin is often less. However, in the presence of precursors such as L-tyrosine or L-DOPA in the growth media, increased production of pyomelanin and eumelanin, respectively, have been reported [16,17]. Literature survey shows that induced-melanin has not been used for mediating the synthesis of nanostructures. In the current investigation, we report (i) enhanced melanin production by resting cells of *Y. lipolytica* NCIM 3590 in the presence of L-DOPA (ii) development of a facile and rapid method for the synthesis of silver and gold nanoparticles using this melanin and (iii) an application of the melanin-mediated silver nanoparticles as effective paint-additives displaying anti-fungal property*.*

## Results and discussion

Melanins are high molecular weight polymers of phenolic compounds that display strong anti-oxidant properties [18-20]. Furthermore, the quinone residues present in melanin are associated with the five-member ring structure that can alternate between the fully-reduced form (phenol form) and the two-electron oxidation product (quinone form) via a semi-quinone state [21]. Such oxidation-reduction reactions involving phenolic compounds are known to mediate nanoparticle synthesis in other biological systems [22]. Taking into account these properties of melanin, we hypothesized that this pigment could be a potential candidate for mediating the reduction of metal salts to their elemental forms as nanostructures. As stated earlier, *Y. lipolytica* has the inherent ability to synthesize melanin that interacts with heavy metals [15]. The low quantity of the cell-associated melanin is often enhanced by incorporation of precursors (L-tyrosine or L-DOPA) [16,17]. In this study, we have over-produced melanin and used this resultant pigment for nanoparticle synthesis.

**Figure 1:**
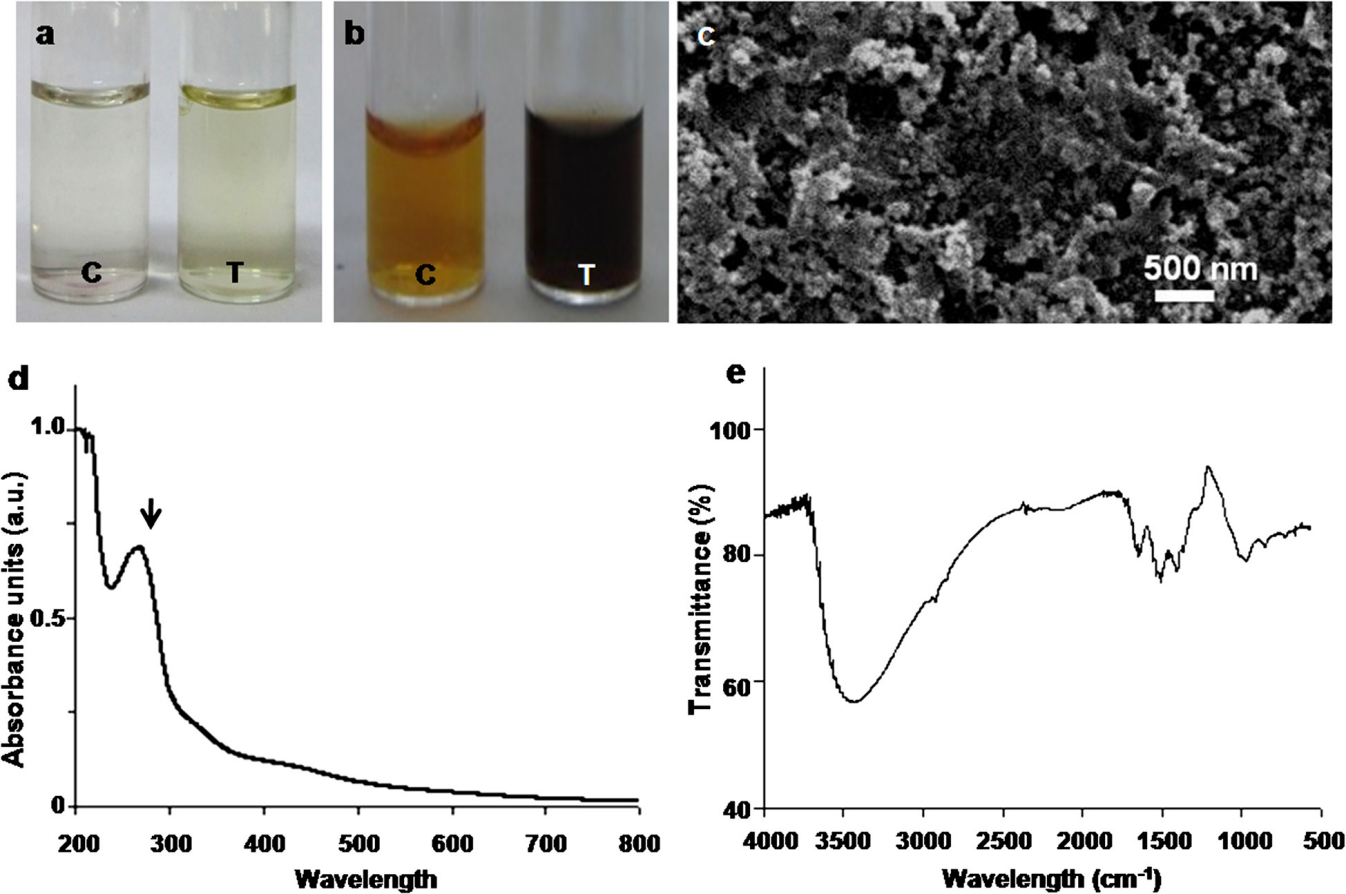
**Characterization of the L-DOPA induced-melanin derived from *Y. lipolytica *NCIM 3590. **Visual observations of reaction mixtures containing (**a**) L-tyrosine (**b**) L-DOPA [in Figures a and b, tube C depicts control tube without cells and tube T depicts reaction mixtures with resting cells]. Representative (**c**) SEM image (**d**) UV-Visible spectrum (**e**) FTIR spectrum.

When washed resting cells of *Y. lipolytica* NCIM 3590 were incubated with L-tyrosine, for 72 h, there was a slight change in color (Figure 1a, Tube T) with respect to the control experiment (Figure 1a, Tube C). The culture was thus not effective in synthesizing L-tyrosine-derived melanin. This is unlike earlier reports on *Y. lipolytica* strain ISA 1668 mediating the synthesis of pyomelanin from L-tyrosine [16,23]. A dark brown color was however observed (Figure 1b, Tube T) when cells were incubated with L-DOPA for shorter time durations (18 h). In contrast, the control mixtures appeared to be light brown (Figure 1b, Tube C). This is in agreement with another report on L-DOPA-mediated induction of eumelanin in *Y. lipolytica* strain CBS 6124 [17]. Thus, precursors such as L-tyrosine or L-DOPA seem to be inducing a strain-specific response with respect to melanin synthesis. In the present study, on scaling up of the process, a maximum yield of 160 mg l^-1^ was obtained. This is comparable with an earlier report on L-tyrosine-mediated melanin production (130 mg l^-1^) by *Klebsiella* sp. GSK [24].

L-DOPA-induced melanin in the current study was isolated by acid precipitation and characterized. The pigment was insoluble in water, ethanol, chloroform and acetone. It was soluble in 1 N NaOH or KOH. The pigment was bleached by NaOCl and H_2_O_2_ as also reported earlier [24,25]. The scanning electron microscope (SEM) images of the preparation showed the presence of nano-scale spherical structures (Figure 1c). The UV- visible spectra displayed a sharp peak at 280 nm (Figure 1d, black arrow) and the absorbance decreased in the visible region which is typical of melanin [24]. Fourier transform infra red (FTIR) spectra (Figure 1e) showed the presence of peaks at 3438 (OH or NH stretching), 1649 (C = C stretching), 1501 (NH bending) and 1415 cm^-1^ (CH_2_-CH_3_ bending) characteristic of this pigment [24,26]. On the basis of the above results we concluded that the pigment was melanin. This melanin derived from *Y. lipolytica* was further used to mediate the synthesis of nanoparticles.

When aliquots of melanin were individually incubated with 1 mM Silver nitrate (AgNO_3_) and chloroauric acid (HAuCl_4_) at 100°C, brown and wine red color, respectively, were observed. Peaks in the UV-visible spectra at around 410 nm indicated the presence of silver nanoparticles and those at 530 nm suggested the synthesis gold nanoparticles. Various conditions influencing melanin-mediated synthesis of nanoparticles were studied. The reaction mixtures were heated at different temperatures, and nanoparticle synthesis was monitored. Incubation at high temperatures enhanced the reaction and the best results were obtained at 100°C. Melanin is a heat-stable compound and protocols for the extraction of this pigment often make use of this property [11,27]. Thus a facile and rapid method for the synthesis of nanoparticle could be developed by using this L-DOPA-induced melanin.

**Figure 2:**
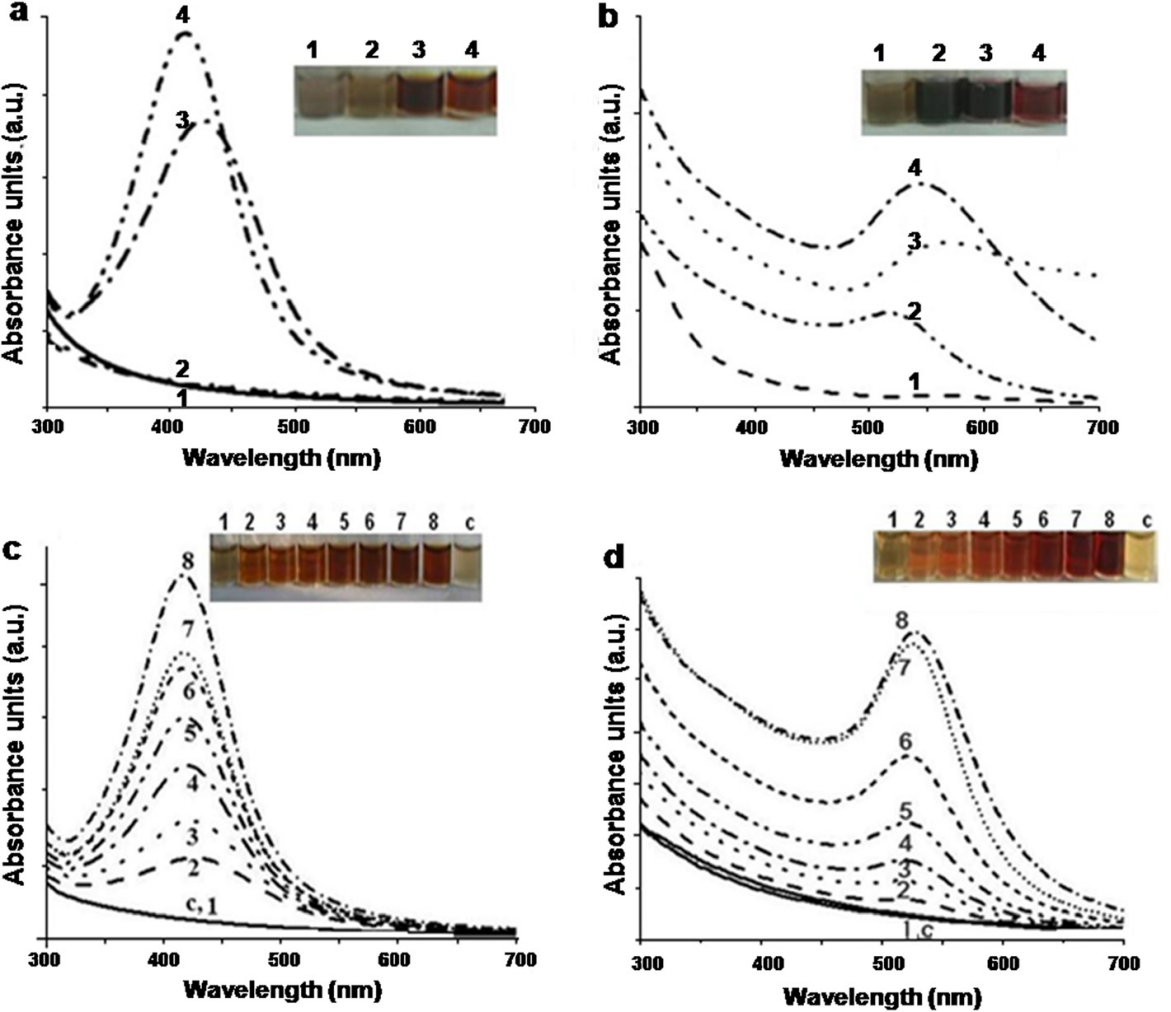
**Effect of pH on the synthesis of (a) silver and (b) gold nanoparticles by L-DOPA induced-melanin derived from *Y. lipolytica *NCIM 3590. **[Reaction mixtures (2 ml) contained 10 μg of melanin at different pH, 1 mM metal salt incubated at 100°C for 10 min. Tubes and lines 1–4 depict pH of 6.0, 8.0, 10.0 and 12.0, respectively]. Effect of salt concentration on the synthesis of (**c**) silver and (**d**) gold nanoparticles [Reaction mixtures (2 ml) contained 10 μg of melanin at pH 12.0 and different concentrations of metal salts incubated at 100°C for 10 min. Tubes and lines 1–8 depict salt concentrations of 0.25, 0.5, 0.75, 1.0, 1.25, 1.5, 1.75 and 2.0 mM, respectively; tube and line C depicts a control without melanin].

Figure 2a and b depict the effect of pH on synthesis of silver and gold nanoparticles, respectively. At pH 6.0 or 8.0, no distinct peaks (around 410 nm) were observed for silver nanoparticles (Figure 2a, lines and tubes 1 and 2). However, under alkaline conditions (pH 10.0 or 12.0), sharp peaks were obtained (Figure 2a, lines and tubes 3 and 4). Similarly, at pH 6.0, gold nanoparticles were not formed (Figure 2b, line and tube 1). As the pH of the reaction mixtures was increased (8.0 and 10.0), peaks indicating the synthesis of gold nanoparticles were observed (Figure 2b, lines and tubes 2 and 3). At pH 12.0, a sharp peak at around 540 nm was obtained (Figure 2b, line and tube 4). Under acidic conditions, melanin is known to aggregate and at high pH, smaller oligomers with a low degree of polymerization are observed. Melanin behaves like a polyelectrolyte, and this property depends on the ionization state of the carboxylic, phenolic and amine groups present in it [27]. The solubility of melanin is high under alkaline conditions [26] and this favored the optimal synthesis of nanostructures.

The effect of silver and gold salt concentration on nanoparticle synthesis is represented in Figure 2c and d, respectively. As the concentration of the salts was progressively increased, the intensity of the respective peaks also increased. Beyond a level of 2 mM (HAuCl_4_ or AgNO_3_) the synthesis was not markedly affected. Such an effect has been reported earlier with other biological material [28,29].

**Figure 3:**
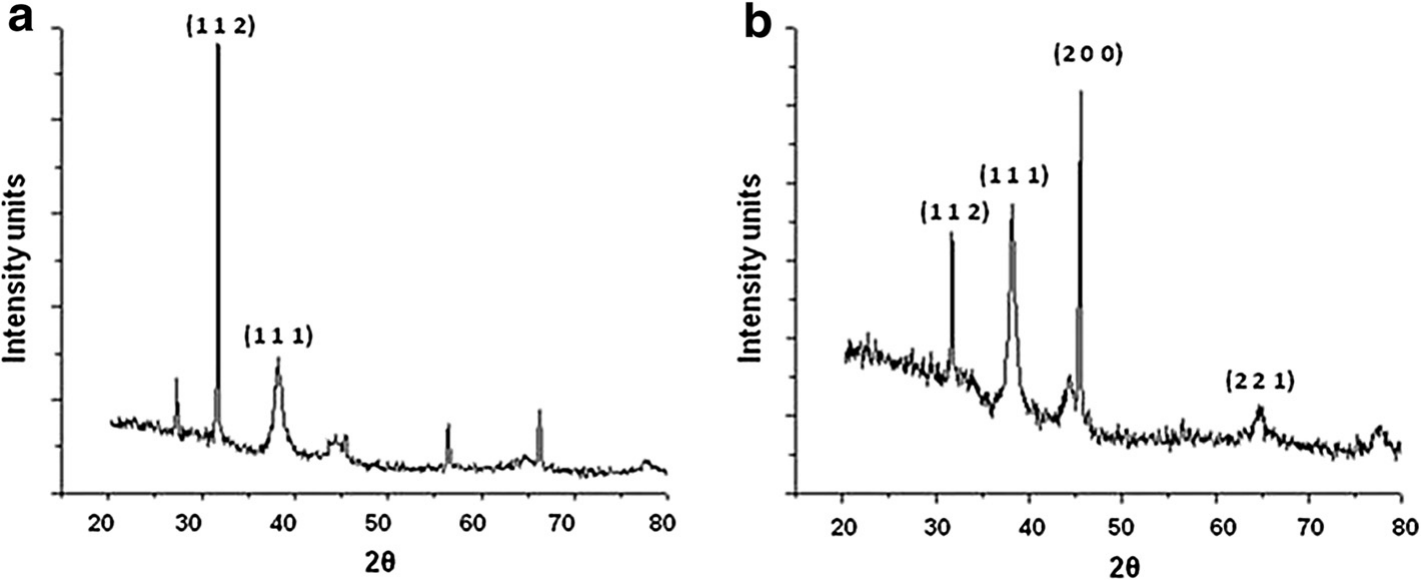
**Representative XRD profiles of melanin-mediated nanoparticles of (a) silver (b) gold.** Three peaks in Figure (**a**) are unassigned.

Figure 3a is a representative X-ray diffraction (XRD) pattern of thin films of silver nanoparticles. The peaks could be indexed to the (1 1 2) and (1 1 1) planes of crystalline silver nanostructures. Biological systems are known to mediate the synthesis of a variety of crystalline silver nanostructures as also reported earlier [30,31]. The XRD patterns for gold nanoparticles showed intense peaks that could be indexed to the (1 1 2), (1 1 1), (2 0 0) and (2 2 1) Bragg’s planes of a faced centre cubic (fcc) lattice structure (Figure 3b). The Bragg reflections in the current investigation are in agreement with earlier reports on gold nanostructures [32].

**Figure 4:**
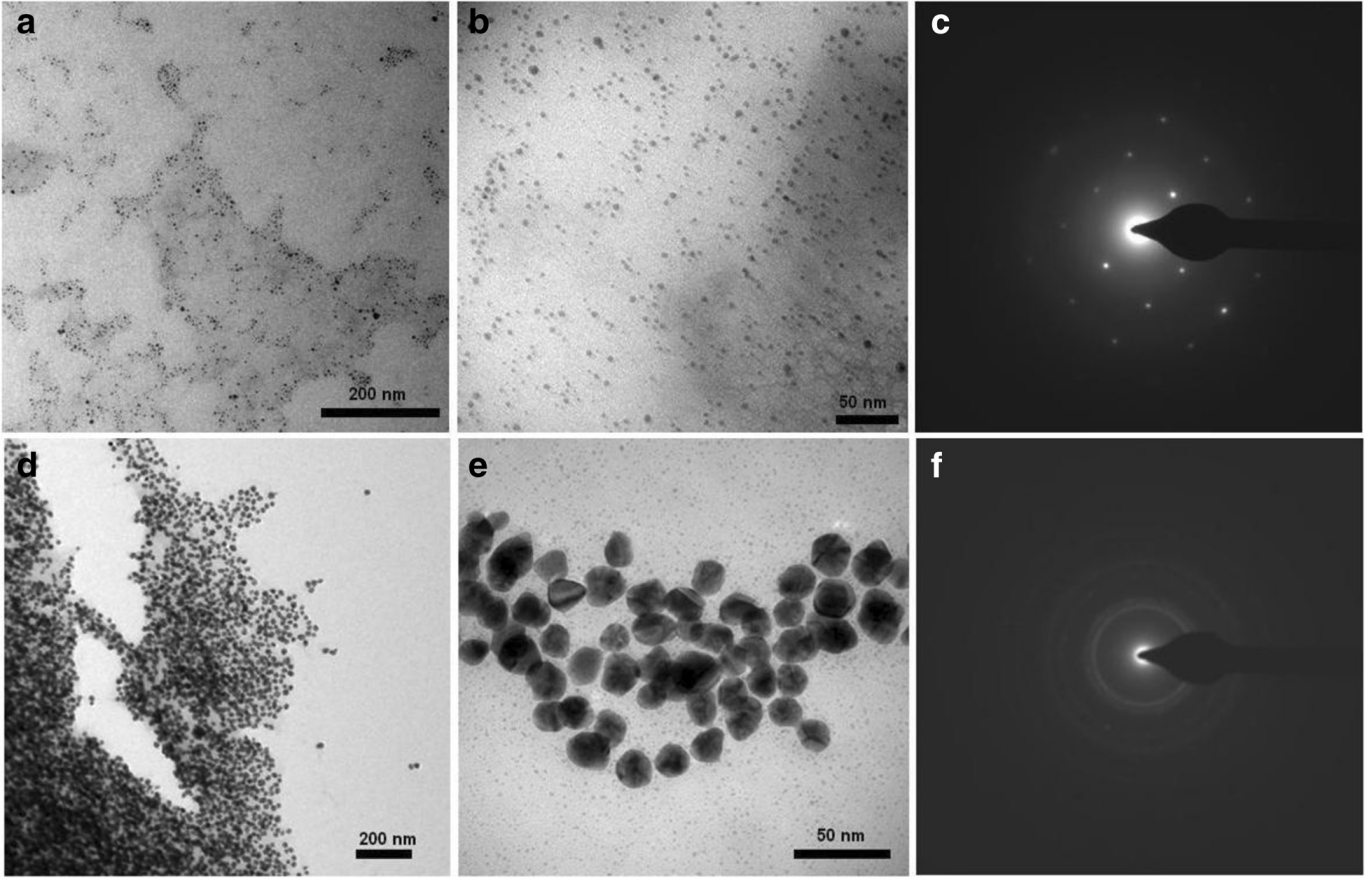
Representative TEM images (at different magnifications) and SAED profiles of melanin-mediated nanoparticles of (a, b and c) silver and (d, e and f) gold, respectively.

**Figure 5:**
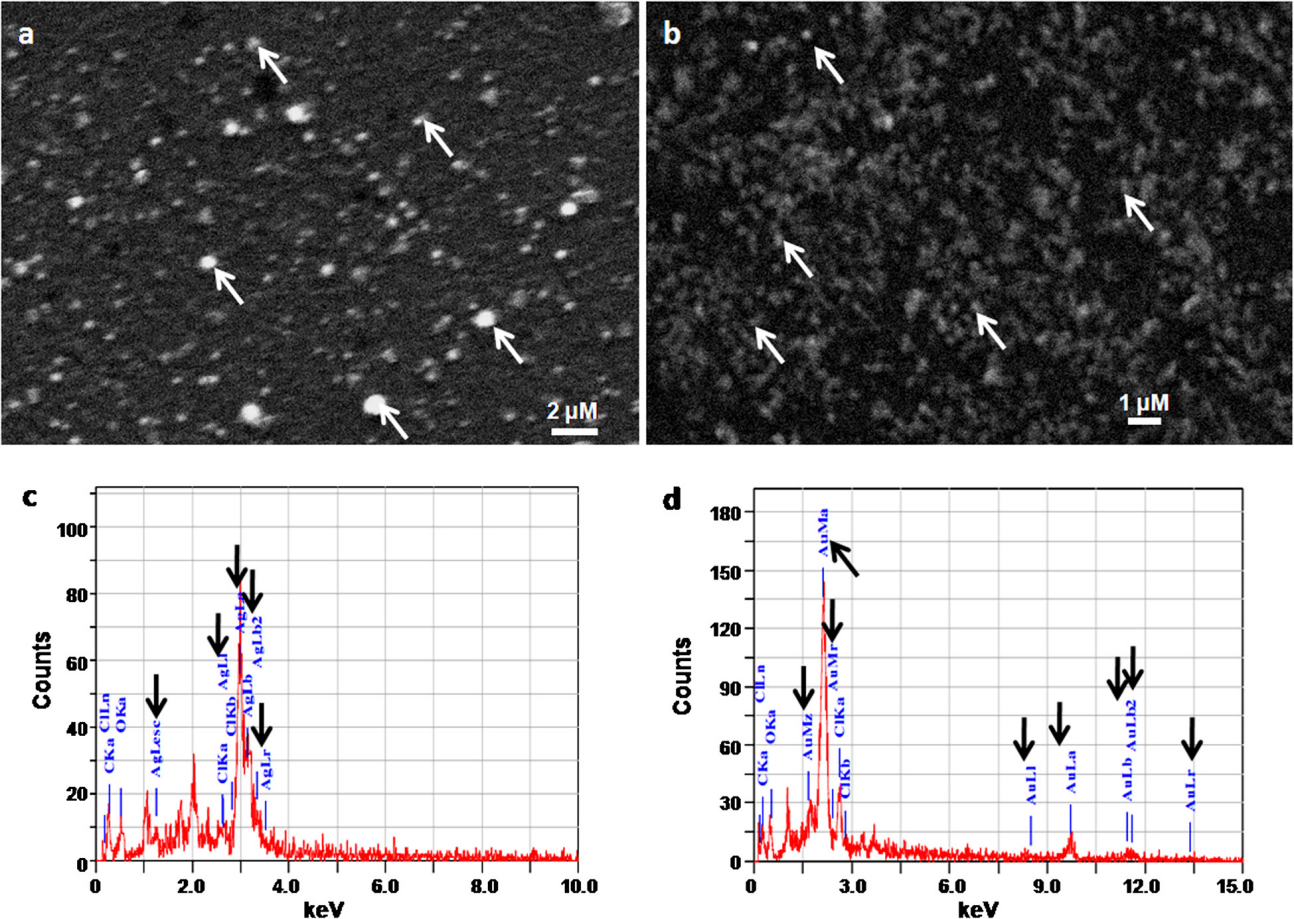
**Representative SEM images of melanin-induced (a) silver and (b) gold nanoparticles. **Representative EDS profiles of (**c**) silver and (**d**) gold nanoparticles.

Both silver as well as gold nanoparticles were fairly mono-disperse with respect to size and shape as evident from the transmission electron microscope (TEM) images (Figures 4a, b, d and e). From these images, the average size of the silver and gold nanoparticles was found to be 7 and 20 nm, respectively. The typical selected area electron diffraction (SAED) patterns are shown in Figures 4c and f. These patterns also confirmed the crystalline nature of the nanoparticles. The structure and composition of the nanoparticles was also determined by Scanning electron microscope-Energy dispersive spectrometer (SEM-EDS) analysis. Representative SEM micrographs of air-dried silver and gold nanostructures on glass surfaces are shown in Figure 5a and b. The nanostructures (Figure 5a and b, white arrows) appeared larger in size when compared to TEM images. Air-drying of the samples on glass slides often results in the aggregation of nanoparticles [29]. Representative EDS profiles for silver and gold nanostructures are shown in Figure 5c and d, respectively. The elemental analysis data and the observed signature spectra (Figure 5c and d, black arrows) confirmed the presence of the specific noble metals in the samples. These results are consistent with earlier reports on the EDS analysis of silver and gold nanostructures synthesized by different biological systems [33,34].

**Figure 6:**
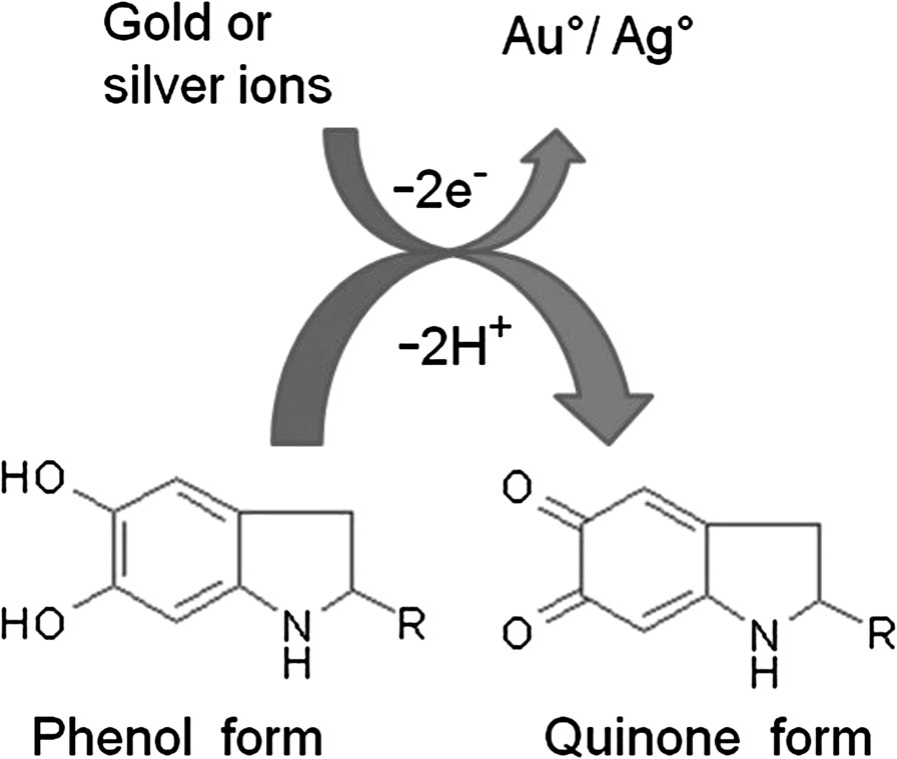
**Proposed mechanism involved in the synthesis of nanoparticles by L-DOPA induced-melanin derived from *Y. lipolytica***.****

FTIR spectra for the control (melanin) and test samples (reaction mixtures with silver and gold nanostructures) were obtained. As also reported earlier with pure melanin [27], peaks for the hydroxyl, amino and aromatic groups were observed. After reaction with the metal salts, the intensities of the bands in different regions were altered. In particular, there was a shift corresponding to the stretching vibration of OH or NH groups and the peaks related to carboxylic group. The absorption peak of the amide-I and -II group also showed a shift. These results indicated the active participation of the aforementioned groups in the process of nanoparticle synthesis. Melanin is known to interact with a variety of transition metals [35]. This decreases the concentration of free metals (and thereby the toxic effect associated with them) or may help in creating a depot of essential metals adjacent to the cell [11]. Melanin acts as an electron exchanger, either oxidizing or reducing metals. Figure 6 depicts the possible mechanism involved in nanoparticle synthesis. The conversion of the hydroxy groups to the quinone groups generates reducing equivalents that are used for the transformation of metal ions into elemental nanostructures.

**Figure 7:**
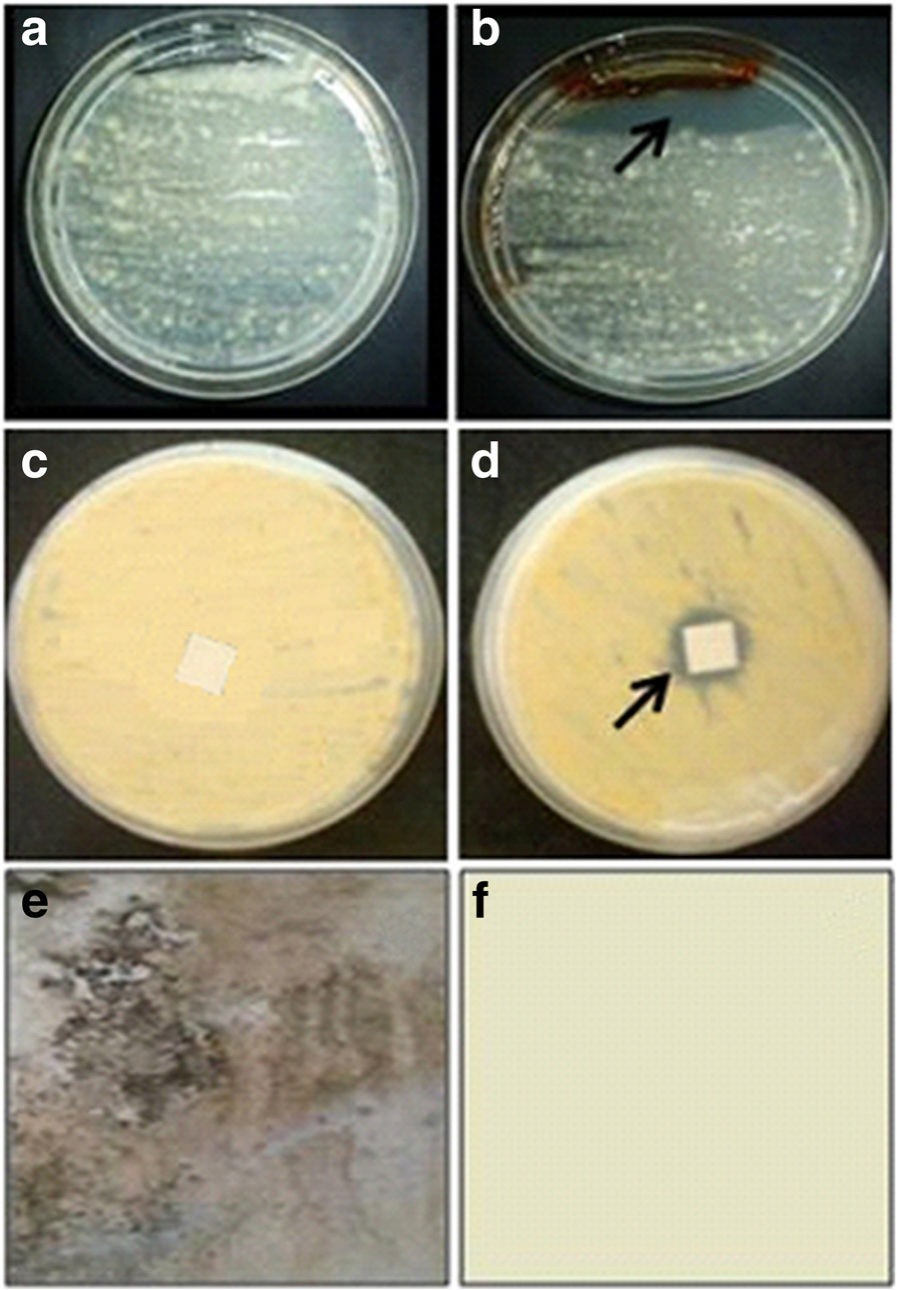
**Anti-fungal activity of silver nanoparticles synthesized by the L-DOPA induced-melanin derived from *Y. lipolytica*. **Plates depicting growth of *Aspergillus *sp. (**a**) without (**b**) with silver nanoparticles. Paint-coated filter paper strips (**c**) without and (**d**) with silver nanoparticles as seen from the base of the plate. Representative pictures depicting the growth of the fungus on glass plates coated with (**e**) un-modified and (**f**) modified paint.

Although there are a large number of reports on the application of silver nanoparticles as anti-bacterial agents [36], there are fewer reports on their anti-fungal properties and applications as paint-additives. Damp painted surfaces often support fungal growth. Bacteria on the other hand, require a higher water activity and do not normally grow on such surfaces. In general, fungi utilize the water-soluble components in the paint for e.g. surfactants, grow rapidly, develop colored spores and cause considerable disfigurement of surfaces [37]. The melanin-mediated silver nanoparticles displaying anti-fungal activity were tested for their effectiveness as a paint-additive. The growth of a fungus (*Aspergillus* sp. isolated from a disfigured wall surface) on a potato dextrose agar (PDA) plate is depicted in Figure 7a. In the experimental plates, silver nanoparticles inhibited the growth of the fungus (Figure 7b, black arrow). Filter paper strips were individually coated with un-modified (without silver nanoparticles) and modified (with silver nanoparticles) paint and placed on the fungus-seeded plates. After incubation for 48 h, the control plates did not show a distinct zone of inhibition (Figure 7c). However, with the silver nanoparticle-coated paper, a marked zone of inhibition was observed (Figure 7d, black arrow). Experimental glass plates were coated with the un-modified and modified paint and subsequently challenged with *Aspergillus* spores. Fungal growth was observed on the plates that were treated with the un-modified paint (Figure 7e). However, when silver nanoparticles were incorporated in the paint, the growth of the fungus was effectively inhibited (Figure 7f). There are reports on the use of “green techniques” for the synthesis of silver-incorporated paints that are effective in preventing the growth of bacteria [38]. In this study, we have reported the effectiveness of silver nanoparticles in preventing the growth of a representative disfigurement causing fungus.

## Conclusions

Melanin is widely distributed in prokaryotic as well as eukaryotic organisms and is known to interact with metals. The use of precursor-induced pigment for synthesizing nanoparticles is being reported here. The conditions for the synthesis of silver and gold nanoparticles have been standardized and the resulting nanostructures have been characterized. An application of the bio-inspired silver nanoparticles as paint-additives with anti-fungal properties (against *Aspergillus* sp.) has also been demonstrated. We thus describe a simple eco-friendly rapid green method for synthesizing nanoparticles of noble metals by using the well-known metal-interacting pigment.

## Methods

### Microorganism, growth and maintenance

A strain of *Y. lipolytica* (NCIM 3590) was used [39]. Stock cultures of the yeast were maintained on YNBG agar slants [yeast nitrogen base (YNB, HiMedia, India): 7.0; dextrose: 10; agar: 20.0 g l^-1^ distilled water]. The cells were grown in YNBG liquid medium (120 rpm, at 20°C, 72 h). After the incubation period, the cells were separated by centrifugation (8000x *g*, 4°C, 10 min) and a fixed number of cells were added during further experiments on melanin synthesis.

### Induction of melanin

Washed cells of *Y. lipolytica* were incubated with L-tyrosine or L-DOPA (SRL, India) and the development of dark color was checked. During these experiments, 10 ml reaction mixtures contained 0.8 ml L-tyrosine or L-DOPA (0.01 mg ml^-1^ in 0.1 N NaOH), 8.8 ml of distilled water and 0.4 ml cell suspension (10^10^ cells ml^-1^). Control tubes without cells were also maintained. The tubes were incubated at 20°C for 18–72 h and the cells were separated by centrifugation (8000x *g*, 4°C, 10 min). The melanin obtained (with L-DOPA as precursor) was isolated by precipitation (with 1 N HCl) and centrifugation (10,000x *g* for 10 min). The black precipitate was washed thrice with distilled water and re-suspended in 0.1 N NaOH. The resulting solution was passed through a 0.22 μ membrane filter and used for further experiments on the characterization and synthesis.

### Characterization of the melanin

The melanin thus synthesized was analyzed by chemical tests such as (i) insolubility in water and common organic solvents (ethanol chloroform and acetone) (ii) solubility in alkaline solutions (1 N NaOH or KOH) and (iii) bleaching with H_2_O_2_ or NaOCl. In addition, the UV-Visible spectra, SEM and FTIR analysis were performed as described later.

### Melanin-mediated synthesis of silver and gold nanoparticles

Silver nitrate, AgNO_3_ (Merck, Germany) and chloroauric acid, HAuCl_4_ (SRL, India) were used. The reaction mixtures contained 1 ml of the isolated melanin (10 μg) and 1 ml of 2.0 mM AgNO_3_ solution unless otherwise mentioned. A similar reaction mixture was used for synthesis of gold nanoparticles using 2.0 mM HAuCl_4_. To enhance the reaction, the mixtures were heated at 100°C for 10 min. To study the effect of temperature on nanoparticle synthesis, the reaction mixtures were incubated at different temperatures (60, 70, 80, 90 or 100°C). The effect of pH was determined by adjusting the reaction mixtures to different pH values (6.0, 8.0, 10.0 or 12.0). The effect of the metal salt concentration was studied by varying the salt content (0.25 to 2.0 mM) in the reaction mixture. All experiments were carried out in triplicates with two biological replicates and representative data is presented here.

### Characterization of the nanoparticles

UV-Visible spectroscopy measurements were performed on a Jasco V-530 spectrophotometer operated at a resolution of 1 nm. For studies on the characterization of nanoparticles, samples from 2 ml reaction mixtures (containing 10 μg of melanin; pH 12.0; 1 mM of metal salts incubated at 100°C for 10 min) were used. XRD measurements of thin films of silver or gold nanoparticles coated on glass slides were carried out in the transmission mode on a D8 Advanced Brucker instrument with Cu Kα radiation using λ = 1.54 A° [28,29].

Samples were immobilized on carbon-coated copper grids (200 μm × 200 μm mesh size) and TEM images were obtained on a TECNAI G2 20U-Twin (FEI, Netherlands) electron microscope, coupled with an energy dispersive X-ray spectrophotometer. SEM observations and elemental analysis were performed on platinum-coated samples that had been previously air-dried on glass slides. An analytical SEM (JEOL JSM-6360A) equipped with EDS was used. All samples were analyzed in triplicates and representative micrographs are included here. To determine the functional groups and their possible role in the synthesis of nanoparticles, FTIR analysis was carried out. The control and test samples were independently blended with potassium bromide to obtain a pellet. The FTIR spectra were collected at resolution of 4 cm^−1^ in the transmission mode (4000–400 cm^−1^) using a Shimadzu FTIR spectrophotometer (FTIR 8400). Shifts in peak maxima in different regions of the spectra were analyzed.

### Anti-fungal activity of silver nanoparticles

Potato dextrose agar (PDA, HiMedia, India) plates were used for testing the anti-fungal properties of the silver nanoparticles. A modified ditch-plate agar method was used. The medium from one end of the agar was marked and removed with a sterile scalpel to make a ditch. A spore suspension containing 10^5^ spores ml^-1^ of *Aspergillus* sp. (isolated from a damp wall surface showing disfigurement) was swabbed on the plate. Silver nanoparticles were added to the ditch. Control plates contained 0.85% saline instead of the silver nanoparticle preparations. The plates were incubated for 48 h at 30°C and fungal growth was monitored.

### Anti-fungal paint assay

Locally available acrylic distemper (un-modified) paint was mixed with the silver nanoparticle preparations (derived from 2 mM silver nitrate) in a proportion of 2:1 (w/w) and was referred to as the ‘modified paint”. The un-modified and modified paints were coated on Whatman no.1 paper strips (1 cm × 1 cm) and air-dried in a laminar flow unit. The coated papers strips were evaluated for anti-fungal properties. These strips were placed on fungus-seeded plates, incubated at 30°C for 48 h and growth inhibition was monitored. Glass plates were coated with un-modified and the modified paint and placed in Petri plates under moist conditions [38]. These were sprayed with spore suspensions of *Aspergillus* sp., a thin layer of PDA medium was added and fungal growth was monitored visually over a period of time.

## Abbreviations

L-DOPA: 3 4-dihydroxy-L-phenylalanine; NCIM: National Collection of Industrial Microorganisms; UV-Visible spectra: Ultra violet-Visible Spectra; XRD: X-ray diffraction; SEM: Scanning electron microscope; FTIR: Fourier transformed infra red; Fcc: Faced centre cubic; TEM: Transmission electron microscope; SAED: Selected area electron diffraction; EDS: Energy dispersive spectrometer; PDA: Potato dextrose agar; YNBG: Yeast nitrogen base glucose.

## Competing interests

The authors declare that they have no competing interests.

## Authors’ contributions

MA and GG performed the experiments, AB and ARK helped with the analysis, MA and SZ wrote manuscript. All authors read and approved the final manuscript.
